# Utility of Serum Ferritin for Predicting Myalgic Encephalomyelitis/Chronic Fatigue Syndrome in Patients with Long COVID

**DOI:** 10.3390/jcm12144737

**Published:** 2023-07-18

**Authors:** Yukichika Yamamoto, Yuki Otsuka, Kazuki Tokumasu, Naruhiko Sunada, Yasuhiro Nakano, Hiroyuki Honda, Yasue Sakurada, Toru Hasegawa, Hideharu Hagiya, Fumio Otsuka

**Affiliations:** Department of General Medicine, Okayama University Graduate School of Medicine, Dentistry and Pharmaceutical Sciences, Okayama 700-8558, Japan; pjqb0kc9@s.okayama-u.ac.jp (Y.Y.); tokumasu@okayama-u.ac.jp (K.T.); pjbc9rog@s.okayama-u.ac.jp (N.S.); y-nakano@okayama-u.ac.jp (Y.N.); ppgf1hrd@okayama-u.ac.jp (H.H.); pzaf6h9w@s.okayama-u.ac.jp (Y.S.); pwcp0od9@okayama-u.ac.jp (T.H.); hagiya@okayama-u.ac.jp (H.H.); fumiotsu@md.okayama-u.ac.jp (F.O.)

**Keywords:** COVID-19, ferritin, insulin-like growth factor-I (IGF-I), long COVID, myalgic encephalomyelitis/chronic fatigue syndrome (ME/CFS)

## Abstract

Objective: The most common symptom of post-acute coronavirus disease 2019 (COVID-19) is fatigue, and it potentially leads to myalgic encephalomyelitis/chronic fatigue syndrome (ME/CFS); however, a specific prognosticator is lacking. We aimed to elucidate the clinical characteristics of patients who developed ME/CFS after COVID-19. Methods: In this retrospective observational study, patients who visited Okayama University Hospital for long COVID between February 2021 and March 2022 were investigated. Results: Of the 234 patients, 139 (59.4%) had fatigue symptoms. Fifty patients with fatigue symptoms (21.4%) met the criteria for ME/CFS (ME/CFS group), while the other 89 patients did not (non-ME/CFS group); 95 patients had no fatigue complaints (no-fatigue group). Although the patients’ backgrounds were not significantly different between the three groups, the ME/CFS group presented the highest scores on the self-rating symptom scales, including the Fatigue Assessment Scale (FAS), EuroQol, and the Self-Rating Depression Scale (SDS). Furthermore, serum ferritin levels, which were correlated with FAS and SDS scores, were significantly higher in the ME/CFS group (193.0 μg/L, interquartile range (IQR): 58.8–353.8) than in the non-ME/CFS group (98.2 μg/L, 40.4–251.5) and no-fatigue group (86.7 μg/L, 37.5–209.0), and a high serum ferritin level was prominent in female patients. Endocrine workup further showed that the ME/CFS group had higher thyrotropin levels but lower growth hormone levels in serum and that insulin-like growth factor-I levels were inversely correlated with ferritin levels (R = −0.328, *p* < 0.05). Conclusions: Serum ferritin level is a possible predictor of the development of ME/CFS related to long COVID, especially in female patients.

## 1. Introduction

Approximately two and a half years have passed since the coronavirus disease 2019 (COVID-19) pandemic broke out. COVID-19 involves both acute and chronic phases. Chronic prolonged symptoms are called post-acute sequelae of severe acute respiratory syndrome coronavirus 2 (SARS-CoV-2) infection, long COVID, or post-COVID-19 condition [1,2,3]. Patients with COVID-19 present with a variety of symptoms which make it difficult for patients to continue their previous social lives for months to years, and some patients have become unable to reintegrate into society after COVID-19 [3]. To respond to society’s needs, we established a COVID-19 aftercare clinic (CAC) to treat patients with long COVID and to comprehend their pathophysiology [4]. The most common symptom in the chronic phase of COVID-19 is fatigue [5,6], and we have discovered that hypogonadism, in addition to thyroidal and adrenal dysfunctions, underlies the long COVID fatigue [7,8,9,10].

Abnormalities in laboratory examinations have been found in some patients with long COVID fatigue, whereas no particular abnormal findings were detected in other patients despite their having long-lasting fatigue. When long COVID is recognized, it has been hypothesized that the pathophysiology of this notorious condition is similar to that of myalgic encephalomyelitis/chronic fatigue syndrome (ME/CFS), post-traumatic stress disorder, or post-viral fatigue syndrome [11,12]. ME/CFS is a debilitating condition with a wide variety of presentations. Diagnosis of ME/CFS is usually based on clinical manifestations (such as fatigue and post-exertional malaise), sleep disturbance, cognitive dysfunction, and orthostatic-related symptoms that persist for at least half a year [13]. Although diseases such as abnormal immune response, infectious disease, and endocrine dysfunction are presumed to be plausible etiologies of ME/CFS, their molecular pathogeneses have not been fully elucidated [14,15]. The prevalence of ME/CFS has been reported to be less than 1% in the general population [16]; however, it ranged up to 16.8% after COVID-19, based on our previous data [9].

There are few objective tests and laboratory examinations that are useful for the diagnosis of ME/CFS; hence, it is very difficult for physicians who are unfamiliar with symptoms of ME/CFS to diagnose the condition [14,17]. In addition, there are no prognostic indicators of long COVID, and there is no established method for identifying patients who may develop ME/CFS after COVID-19 [9,14]. In this study, we investigated patients who developed ME/CFS after COVID-19 and attempted to elucidate the clinical characteristics of related laboratory parameters.

## 2. Patients and Methods

### 2.1. Study Design and Patients

This retrospective observational study was performed at a single center in Japan. Patients who visited the CAC at Okayama University Hospital for prolonged symptoms after suffering COVID-19 between 22 February 2021 and 31 May 2022 were eligible. We enrolled patients who had persistent symptoms for more than 4 weeks after COVID-19 onset before 30 November 2021 and without obviously pre-existing hyperferritinemia. The patients were categorized into the following three groups: (1) an “ME/CFS group”, which included patients with a clinical diagnosis of ME/CFS based on all of the three internationally standardized criteria, including the Fukuda criteria [18], the Canadian Consensus Criteria (CCC) [19], and the Institute of Medicine criteria (IOM) [20]; (2) a “non-ME/CFS group”, which included patients who had a complaint of fatigue but did not meet the ME/CFS criteria; and (3) a “no-fatigue group”, which included patients without any fatigue.

### 2.2. Clinical Characteristics

We reviewed the medical records of the patients in June 2022 and obtained information about sex, age, body mass index (BMI), number of days from onset of COVID-19 to the first visit to the CAC, acute-phase severity, and care for COVID-19. The severity of chronic-phase symptoms at the first visit to the CAC was assessed using the Fatigue Assessment Scale (FAS) [21,22], EuroQol 5 Dimensions 5 Levels (EQ-5D-5L) [23], the Self-Rating Depression Scale (SDS) [24,25], and the Frequency Scale for the Symptoms of Gastroesophageal Reflux Disease (FSSG) [26]. Information on the patients’ vital signs and results of laboratory examinations, including blood cell count, biochemistry, and endocrine parameters, was obtained at the first visit to the CAC.

### 2.3. Laboratory Examinations

Blood sampling was performed in a relaxed sitting position at the time each patient visited the CAC, which was around late morning to early afternoon. The blood tests were determined by each physician, and the measurements were performed using the Cobas 8000 auto-analyzer system (F. Hoffmann-La Roche AG, Basel, Switzerland) at the central laboratory of our facility. Assays for serum ferritin were performed by an electro-chemiluminescence immunoassay (ECLIA) using the Elecsys Ferritin kit (F. Hoffmann-La Roche AG). Assays for plasma adrenocorticotropin (ACTH) and serum cortisol were also performed by ECLIA using Elecsys ACTH and Elecsys Cortisol II kits (F. Hoffmann-La Roche AG), respectively, as previously reported [8]. Assays for serum free thyroxin (FT4) and thyrotropin (TSH) were performed using Elecsys FT4 III and TSH kits, and assays for serum growth hormone (GH) and insulin-like growth factor (IGF)-I were performed using Elecsys GH and IGF-I kits (F. Hoffmann-La Roche AG), respectively.

### 2.4. Statistical Analysis

EZR, version 1.55 (Saitama Medical Center, Jichi Medical University, Saitama, Japan), a graphical user interface for R (The R Foundation for Statistical Computing, Vienna, Austria), was used for all statistical analyses [27]. The data were analyzed using Fisher’s exact test and the Kruskal–Wallis test to compare categorical variables. If the null hypothesis was rejected by the Kruskal–Wallis test, the Steel–Dwass test was added. Pearson’s correlation coefficient was used to statistically analyze continuous measurements. Statistical significance was defined as * *p* < 0.05 and ** *p* < 0.01.

### 2.5. Ethical Approval

Patients who wished to opt out were given the opportunity to do so after being informed about the study on our hospital’s wall and website. This study was approved by the Okayama University Hospital Ethics Committee (no. 2105-030) and adhered to the Declaration of Helsinki. Informed consent from the patients was not necessary owing to the anonymization of the data. All authors had access to the study data and reviewed and approved the final manuscript.

## 3. Results

Of the 312 patients who visited the CAC during the study period, 78 patients did not meet the inclusion criteria. These 78 patients included 2 patients who visited the CAC less than 4 weeks after the onset of COVID-19, 75 patients whose disease onset was before 30 November 2021, and 1 patient who had hyperferritinemia prior to suffering from COVID-19. Data from the remaining 234 patients were analyzed in this study. All of these patients were followed up for at least 6 months from the onset of the disease. While 95 patients (40.6%) did not complain of general fatigue, the remaining 139 patients (59.4%) complained of general fatigue. Of the patients with fatigue symptoms, 50 patients (40.0%) met all of the three sets of diagnostic criteria for ME/CFS (Fukuda criteria, CCC, and IOM criteria), and these patients were included in the ME/CFS group, whereas the remaining 89 patients with fatigue were included in the non-ME/CFS group.

### 3.1. Patients’ Backgrounds

The backgrounds of the patients, including age, BMI, number of days from the onset of COVID-19 to the first visit to the CAC, acute-phase treatments for COVID-19, and vital signs on admission, are summarized in Table 1. The ME/CFS group included 24 males (48.0%) and 26 females (52.0%) with a median age of 42 years (interquartile range (IQR): 30.3–51.8). Sex, age, and BMI were not significantly different between the three groups. The median duration from the onset of COVID-19 to the first visit to the CAC was 128 days (IQR: 72.3–205.5) in the ME/CFS group, and it was significantly longer than that in the non-ME/CFS group (median: 73 days, IQR: 55.0–114.0) and that in the no-fatigue group (median: 106 days, IQR: 73.0–152.0; ** *p* < 0.01). The variables of acute-phase treatments, including place of care and oxygen or steroid administration, were not significantly different between the three groups. Systolic blood pressure was slightly, but not significantly, higher in the ME/CFS group (median: 129 mmHg, IQR: 112–140) than in the other two groups.

### 3.2. Severity of Fatigue-Related Symptoms

**Figure 1:**
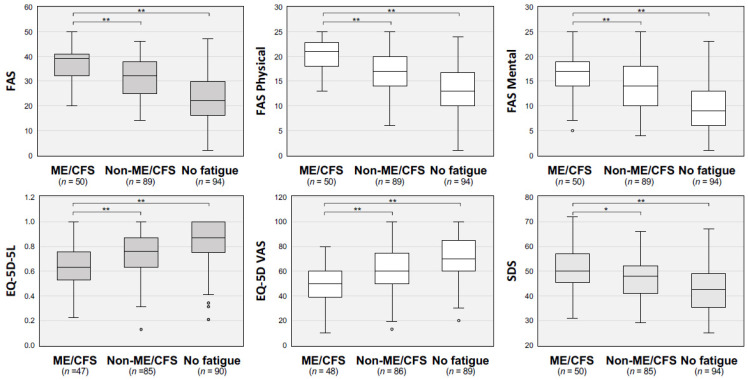
Self-rating scales in ME/CFS, non-ME/CFS, and no-fatigue groups of patients with long COVID. Self-rating symptom scales of each group, including fatigue assessment scale (FAS) of physical and mental evaluation, self-rating depression scale (SDS), EuroQol 5 Dimensions (EQ-5D) and 5 Levels (EQ-5D-5L), and EQ-5D visual analog scale (EQ-5D-VAS), were compared. The median is represented by the horizontal bar within the box, and the upper and lower horizontal lines of the box represent the 75th and 25th percentiles, respectively. The upper and lower horizontal bars outside the box represent the maximum and minimum values within 1.5 times the interquartile range, respectively, and the circles outside indicates outliers. The Kruskal–Wallis test was performed for statistical analysis, and * *p* < 0.05 and ** *p* < 0.01 indicate statistically significant differences between the indicated groups.

The scores of the self-rating scales, including FAS, FAS physical, FAS mental, EQ-5D-5L, EQ-5D VAS, and SDS, are shown in Figure 1. The ME/CFS group had significantly more severe scores for all self-rating scales than those in the other two groups.

### 3.3. Laboratory Data and Correlations with the Self-Rating Scales

**Figure 2:**
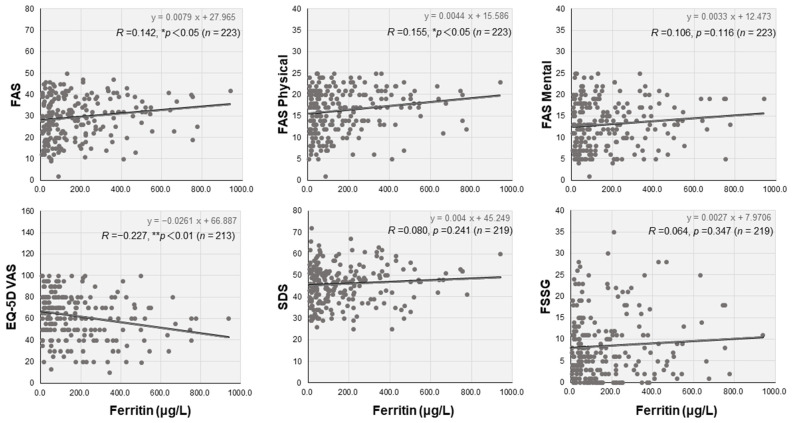
Interrelationships between the levels of serum ferritin and self-rating scales in patients with long COVID. Scatter diagrams between serum ferritin and self-rating symptom scales, including FAS, SDS, EQ-5D-5L, EQ-5D-VAS, and frequency scale for symptoms of gastroesophageal reflux disease (FSSG), are shown (see Figure 1 legend). The interrelationships between serum ferritin levels and these scores were analyzed using Pearson’s correlation coefficient, and * *p* < 0.05 and ** *p* < 0.01 indicate statistically significant correlations.

**Figure 3:**
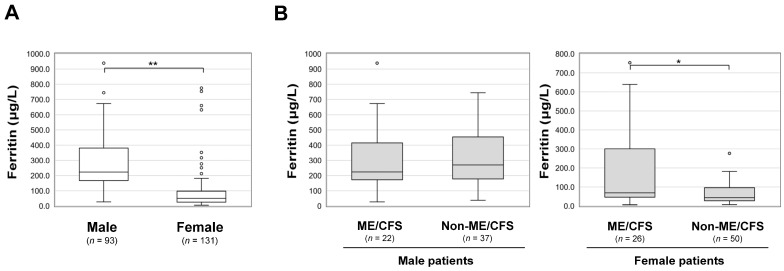
Sex-related differences in the levels of serum ferritin in patients with long COVID. (**A**) The serum ferritin levels were compared in male and female patients with long COVID. (**B**) Serum ferritin levels in male and female patients were individually compared between the ME/CFS and non-ME/CFS groups. The details of each panel are shown in the legend of Figure 1. Fisher’s exact test was performed for statistical analysis, and * *p* < 0.05 and ** *p* < 0.01 indicate statistically significant differences.

Blood cell counts and biochemical data were comprehensively analyzed in the three groups (Table 2). Of note, serum ferritin levels were independently and significantly higher in the ME/CFS group (193.0 μg/L, IQR: 58.8–353.8) than in the non-ME/CFS group (98.2 μg/L, IQR: 40.4–251.5) and the no-fatigue group (86.7 μg/L, IQR: 37.5–209.0) (* *p* < 0.05). However, there were no differences in other parameters, including hemoglobin, inflammatory values, and coagulation markers, among the three groups. Interestingly, serum ferritin levels were weakly but positively correlated with FAS (*R =* 0.142, * p < 0.05) and FAS Physical (*R =* 0.155, * p < 0.05) and negatively correlated with EQ-5D VAS scores (*R =* −0.227, ** p < 0.01), but they were not correlated with FAS Mental, SDS, or FSSG scores (Figure 2). Serum ferritin levels were significantly lower in women (50.3 μg/L, IQR: 25.6–97.3) than in men (223.0 μg/L, IQR: 167.0–381.0; ** *p* < 0.01). Hyperferritinemia was significantly higher in the ME/CFS group (68.9 μg/L, IQR: 46.4–300.5) than in the non-ME/CFS group (43.8 μg/L, IQR: 27.1–96.2) in female patients (* *p* < 0.05), although the difference was not significant in male patients (Figure 3).

### 3.4. Endocrine Characteristics and Correlations with Serum Ferritin Levels

Table 3 shows the hormonal parameters for the three groups. Serum cortisol levels, plasma ACTH levels, and ACTH/cortisol ratios were not significantly different between the three groups. Serum TSH levels were significantly higher in the ME/CFS group (1.37 μIU/mL, IQR: 0.99–2.01) than in the non-ME/CFS group (1.06 μIU/mL, IQR: 0.80–1.56; * *p* < 0.05). Serum FT4 levels did not differ among the three groups. The FT4/TSH ratio was significantly lower in the ME/CFS group (0.87, IQR: 0.64–1.22) than in the non-ME/CFS group (1.15, IQR: 0.83–1.69; * *p* < 0.05). Serum GH levels in the ME/CFS group (0.22 ng/mL, IQR: 0.05–0.67) were significantly lower than in the no-fatigue group (0.37 ng/mL, IQR: 0.17–1.11; ** *p* < 0.01).

**Figure 4:**
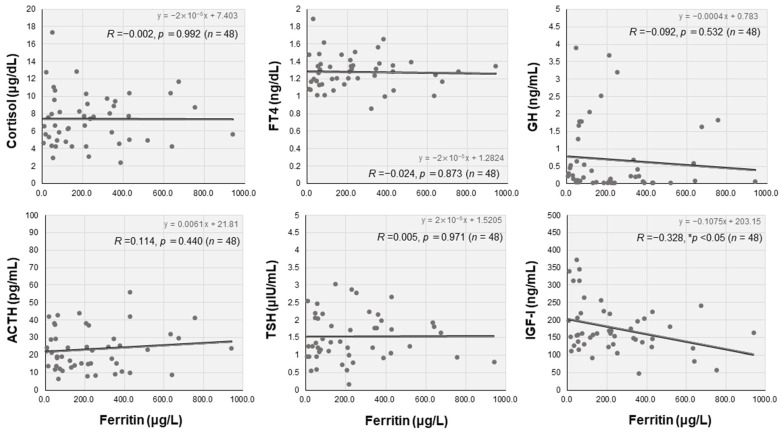
Interrelationships between levels of serum ferritin and key endocrine markers in the ME/CFS group. The scatter diagrams show levels of serum ferritin and endocrine markers, including cortisol, adrenocorticotropin (ACTH), free thyroxine (FT4), growth hormone (GH), insulin-like growth factor (IGF)-I, and thyrotropin (TSH), in the ME/CFS group. These interrelationships were analyzed using Pearson’s correlation coefficient, and * *p* < 0.05 indicates statistically significant correlations.

We also analyzed the relationships between hormonal parameters and serum ferritin levels. Serum IGF-I levels were significantly and inversely correlated with serum ferritin levels in the ME/CFS group (*R =* −0.328, * p < 0.05) (Figure 4). Furthermore, the correlation between serum IGF-I and ferritin levels was stronger in the female patients in the ME/CFS group (*R =* −0.497, ** p < 0.01).

## 4. Discussion

Our study revealed that serum ferritin levels were about two times higher in patients with long COVID (particularly in women) who thereafter developed ME/CFS than in patients with long COVID who did not develop ME/CFS. Ferritin synthesis is regulated by oxidation and anti-oxidation and is essential for in vivo inflammation and iron metabolism [28]. Hyperferritinemia is caused by hemochromatosis, inflammatory diseases, infectious diseases, hematological and malignant diseases, and liver and renal dysfunctions. According to an epidemiological study, the most common cause was infectious disease, followed by solid tumors and liver dysfunction [28,29,30].

ME/CFS has been hypothesized to develop under the condition of abnormal immune responses, viral infection, enhancement of oxidative and nitrosative stress pathways, or dysfunction of the hypothalamic–pituitary–adrenal (HPA) axis [31,32,33]. Possible pathological mechanisms of long COVID include immune system abnormalities, direct viral tissue damage, and endocrine disability [34,35,36,37]. With regard to immune abnormalities, studies have focused on several cytokines. The hypothesis of immune abnormalities as ME/CFS pathomechanisms has previously led to research on immune abnormalities in ME/CFS. Although some biomarkers, including antibodies against β-adrenergic receptors and muscarinic acetylcholine receptors, oxidative stress and anti-oxidative activity, and inflammatory cytokines, such as interleukin-1β, tumor necrosis factor-α, and nuclear factor-kappa B, have been studied, none of them has sufficient reliability for clinical use in the diagnosis or severity evaluation of ME/CFS [14,38,39]. The levels of serum ferritin have been reported to be elevated in patients with ME/CFS, as in our study, but the evidence is controversial [40]. Since iron deficiency can cause fatigue symptoms and mimic ME/CFS, it is recommended to exclude hypoferritinemia when observing patients with ME/CFS in primary care settings [41,42]. Female patients are more likely to suffer from iron deficiency and show lower serum ferritin levels than male patients [30]. Therefore, our results showing that serum ferritin levels were considerably higher in female patients with long COVID in the ME/CFS group, who would normally have lower ferritin levels, are clinically important.

In addition to the physiological elevation of serum ferritin levels associated with infection, the acute phase of COVID-19 causes localized inflammation of the lungs, leading to a systemic and extrapulmonary hyperinflammation syndrome and the production of excessive serum ferritin [43]. Hence, serum ferritin levels have been reported to reflect the inflammatory response and are useful markers for diagnosing and predicting the severity of the disease [44,45]. In contrast, in the chronic phase of COVID-19, hyperferritinemia has also been suggested to be a clinical feature of long COVID, as shown in our study, though the number of studies has been limited [46,47,48]. However, we cannot simply explain the phenomenon of hyperferritinemia as prolonged inflammation induced by the acute phase of COVID-19. This is because serum ferritin levels were only elevated in patients with ME/CFS without any other inflammatory markers, despite the longer time since the onset of COVID-19. In addition, no specific relationship between severity of the acute phase and development of ME/CFS was found.

In the chronic phase of COVID-19, hyperferritinemia may be caused by iron dyshomeostasis [49]. As an example of the possible mechanism in ME/CFS patients, hepcidin-like effects might be involved in this mechanism if SARS-CoV-2 specifically elevates ferritin levels in the context of direct and indirect metabolic involvement by viral infection [50]. In addition, hyperferritinemia in long COVID may be related to accompanying hypertension, sleep disturbances, or depression, all of which are consistent with the results of our studies showing that blood pressure and SDS were higher in patients with ME/CFS [46,47,51].

In the present study, we also uncovered a relationship between hyperferritinemia and endocrine profiles among patients with ME/CFS after COVID-19. Endocrine dysfunction, including hypothyroidism and impaired HPA axis, has been reported in patients with ME/CFS [32,52]. However, patients in the ME/CFS group in our study only had a tendency for occult hypothyroidism, and there was no significant difference in HPA axis function between the ME/CFS group and the other two groups. These findings may reflect the underlying mechanism specific to long COVID, and further investigation of the relationship between ferritin levels and hormonal characteristics showed a negative correlation between serum ferritin and serum IGF-I levels. IGF-I, which plays an important role in childhood growth and anabolism, also regulates ferritin synthesis at the messenger ribonucleic acid level [53,54]. Furthermore, IGF-I is known to be transiently downregulated in response to various stresses, malnutrition, and depression [55,56]. Taken together, serum IGF-I levels may be reduced by various factors associated with COVID-19, which may result in secondary dysregulation of serum ferritin levels in patients with long COVID. According to our study, this tendency was more prominent in female patients, suggesting that the presence of hyperferritinemia should be considered for ME/CFS, especially in female patients with post-COVID-19.

Our study had several limitations. First, all of the patients were referred from other hospitals; therefore, this study included only patients with more severe conditions. Second, we did not clearly distinguish patients who already had ME/CFS at the time of the visit to the CAC from those who developed ME/CFS over the time course. Both sets of patients were evaluated, and their blood samples were collected at the same time as the first visit. Third, since this study was a cross-sectional observational study without any follow-up, there may have been causal reversals, and the trends in serum ferritin levels could not be observed. Further prospective studies are warranted to validate the results. Fourth, monovariate analysis was repeated without adjustment, so a type I error could not be completely avoided. Fifth, our study was performed at a single center only with long COVID patients. A multicenter study with non-long COVID patients as controls is desired.

In conclusion, we revealed the clinical characteristics of patients who developed ME/CFS after COVID-19 and identified serum ferritin as a possible predictor of ME/CFS related to long-term COVID. Hyperferritinemia was objectively correlated with the severity of long-term COVID symptoms as well as endocrine hormone secretion. However, the mechanism remains hypothetical, and further in vivo and in vitro investigations are necessary to understand the characteristics of ferritin metabolism in patients with ME/CFS and long COVID.

## Figures and Tables

**Table 1:** Background information of patients with long COVID with or without symptoms of ME/CFS.

	ME/CFS (*n* = 50)	Non-ME/CFS (*n* = 89)	No Fatigue (*n* = 95)	*p*-Value
Characteristics				
Sex: male/female	24/26	37/52	38/57	0.482 ^(a)^
Age (years), median (IQR)	42 (30.3–51.8)	39 (24.0–50.0)	43 (29.5–51.0)	0.519 ^(b)^
BMI (kg/m^2^), median (IQR)	24.7 (21.3–27.4)	23.4 (20.7–25.7)	22.7 (19.8–27.0)	0.326 ^(b)^
Days from the onset to the first visit, median (IQR)	128 (72.3–205.5) ^☨^	73 (55.0–114.0) ^☨^	106 (73.0–152.0)	<0.01 ^(b)^
Acute COVID-19 treatments				
At home (%)	17 (34.0)	35 (39.3)	45 (47.4)	0.587 ^(a)^
At accommodation facilities (%)	15 (30.0)	35 (39.3)	27 (28.4)	0.357 ^(a)^
Hospitalized (%)	22 (44.0)	27 (30.3)	30 (31.6)	0.139 ^(a)^
Oxygen therapy (%)	10 (20.0)	12 (13.5)	16 (16.8)	0.339 ^(a)^
Steroid (%)	12 (24.0)	13 (14.6)	13 (14.5)	0.176 ^(a)^
Vital signs				
Systolic blood pressure (mmHg), median (IQR)	129 (112–140)	120 (107–130)	120 (108–137)	0.068 ^(b)^
Diastolic blood pressure (mmHg), median (IQR)	74 (66–82)	73 (61–81)	69 (63–80)	0.581 ^(b)^
Heart rate (bpm), median (IQR)	82 (75–89)	81 (73–91)	79 (73–89)	0.627 ^(b)^

BMI, body mass index; IQR, interquartile range. Data in the ME/CFS, non-ME/CFS, and no-fatigue groups were analyzed using ^(a)^ Fisher’s exact test or ^(b)^ the Kruskal–Wallis test. Differences were considered statistically significant at *p* < 0.05. ^☨^: Statistically significant between the indicated groups.

**Table 2:** Laboratory data of ME/CFS, non-ME/CFS, and no-fatigue groups.

	ME/CFS (*n* = 48)	Non-ME/CFS (*n* = 87)	No Fatigue (*n* = 89)	*p*-Value
Blood cell counts				
WBC (×10^3^/μL)	6.62 (5.32–7.44)	5.89 (4.77–7.09)	6.01 (4.75–7.58)	0.560
RBC (×10^6^/μL)	4.72 (4.42–5.00)	4.54 (4.23–4.94)	4.51 (4.14–4.91)	0.187
Hb (g/dL)	14.70(13.9–15.7)	14.3 (13.1–15.3)	14.1 (13.0–15.2)	0.091
Plt (×10^3^/μL)	265.0 (215.3–308.3)	262.0 (236.0–314.5)	253.5 (228.5–291.8)	0.611
Biochemistry				
TP (g/dL)	7.2 (7.0–7.5)	7.1 (6.9–7.5)	7.1 (6.9–7.4)	0.284
Alb (g/dL)	4.5 (4.3–4.7)	4.4 (4.2–4.6)	4.4 (4.1–4.6)	0.083
T-Bil (mg/dL)	0.72 (0.48–0.89)	0.60 (0.49–0.83)	0.60 (0.47–0.82)	0.368
AST (U/L)	19.5 (15.0–25.0)	19.0 (15.0–24.0)	19.0 (16.0–23.0)	0.952
ALT (U/L)	17.0 (12.8–33.3)	16.0 (11.5–29.5)	17.0 (11.0–27.3)	0.505
ALP (U/L)	68.0 (58.3–84.0)	65.0 (59.5–82.5)	69.0 (58.0–94.3)	0.632
CK (U/L)	75.0 (58.8–111.0)	72.0 (56.5–97.5)	77.0 (59.0–112.0)	0.522
UN (mg/dL)	12.7 (10.5–14.7)	11.5 (10.0–13.8)	12.9 (11.0–15.5)	0.086
Cr (mg/dL)	0.73 (0.60–0.83)	0.69 (0.59–0.80)	0.67 (0.59–0.77)	0.487
LDL-C (mg/dL)	118.5 (102.0–146.3)	117.0 (99.5–149.5)	123.0 (88.0–138.0)	0.625
BS (mg/dL)	99.0 (93.8–108.3)	100.0 (90.0–110.0)	101.0 (91.3–115.0)	0.767
CRP (mg/dL)	0.06 (0.03–0.10)	0.06 (0.03–0.14)	0.07 (0.02–0.16)	0.770
ESR (mm/hr)	7.0 (3.5–11.3)	9.0 (5.0–17.0)	8.0 (5.0–14.0)	0.086
Ferritin (μg/L)	193.0 (58.8–353.8) ^☨^	98.2 (40.4–251.5)	86.7 (37.5–209.0) ^☨^	<0.05
IgG (mg/dL)	1193.9 (1075.2–1283.1)	1188.8 (1061.2–1353.6)	1258.3 (1055.6–1391.2)	0.533
Fibrinogen (mg/dL)	282 (256–308)	298 (257–348)	288 (253–348)	0.194
D-dimer (μg/L)	0.5 (0.5–0.5)	0.5 (0.5–0.5)	0.5 (0.5–0.5)	0.353

Alb, albumin; ALT, alanine aminotransferase; ALP, alkaline phosphatase; AST, aspartate aminotransferase; BS, blood sugar; CK, creatine kinase; Cr, creatinine; CRP, C-reactive protein; ESR, erythrocyte sedimentation rate; Hb, hemoglobin; IgG, immunoglobulin G; LDL-C, low-density lipoprotein cholesterol; Plt, platelets; RBC, red blood cells; T-Bil, total bilirubin; TP, total protein; UN, urea nitrogen; WBC, white blood cells. The data in the ME/CFS, non-ME/CFS, and no-fatigue groups were analyzed using the Kruskal–Wallis test. Differences were considered statistically significant at *p* < 0.05. ^☨^: Statistically significant between the indicated groups.

**Table 3:** Endocrine data of ME/CFS, non-ME/CFS, and no-fatigue groups.

	ME/CFS (*n* = 48)	Non-ME/CFS (*n* = 87)	No Fatigue (*n* = 89)	*p*-Value
Endocrine data				
Cortisol (μg/dL)	7.1 (5.0–9.3)	7.8 (5.4–10.2)	6.5 (4.2–8.6)	0.102
ACTH (pg/mL)	22.2 (14.1–29.6)	20.5 (14.9–28.5)	19.6 (13.9–25.6)	0.598
FT4 (ng/dL)	1.28 (1.16–1.36)	1.26 (1.17–1.41)	1.24 (1.14–1.35)	0.426
TSH (μIU/mL)	1.37 (0.99–2.01) ^☨^	1.06 (0.80–1.56) ^☨^	1.41 (1.01–2.08)	<0.05
GH (ng/mL)	0.22 (0.05–0.67) ^☨^	0.19 (0.07–0.66)	0.37 (0.17–1.11) ^☨^	<0.01
IGF-I (ng/mL)	161 (132–211)	145 (122–191)	139 (104.0–199.0)	0.121
ACTH/Cortisol	3.32 (2.09–4.57)	2.86 (2.03–4.02)	3.30 (2.43–4.59)	0.348
FT4/TSH	0.87 (0.64–1.22) ^☨^	1.15 (0.83–1.69) ^☨^	0.85 (0.56–1.34)	<0.05

ACTH, adrenocorticotropin; FT4, free thyroxine; GH, growth hormone; IGF-I, insulin-like growth factor-I; TSH, thyrotropin. The data in the ME/CFS, non-ME/CFS, and no-fatigue groups were analyzed using the Kruskal–Wallis test. We regarded *p* < 0.05 or *p* < 0.01 as statistically significant differences. ^☨^: Statistically significant difference between the indicated groups.

## Data Availability

The datasets generated and analyzed in the present study are available from the corresponding authors on reasonable request.
